# Integrated Analysis of Energy Metabolism Signature-Identified Distinct Subtypes of Bladder Urothelial Carcinoma

**DOI:** 10.3389/fcell.2022.814735

**Published:** 2022-02-23

**Authors:** Fan Zhang, Jiayu Liang, Dechao Feng, Shengzhuo Liu, Jiapei Wu, Yongquan Tang, Zhihong Liu, Yiping Lu, Xianding Wang, Xin Wei

**Affiliations:** ^1^ Department of Urology, Institute of Urology, West China Hospital, Sichuan University, Chengdu, China; ^2^ Department of Pediatric Urology, West China Hospital, Sichuan University, Chengdu, China

**Keywords:** bladder cancer, prognosis, metabolic status, nonogram, signature

## Abstract

**Background:** Bladder urothelial carcinoma (BLCA) is the most common type of bladder cancer. In this study, the correlation between the metabolic status and the outcome of patients with BLCA was evaluated using data from the Cancer Genome Atlas and Gene Expression Omnibus datasets.

**Methods:** The clinical and transcriptomic data of patients with BLCA were downloaded from the Cancer Genome Atlas and cBioPortal datasets, and energy metabolism-related gene sets were obtained from the Molecular Signature Database. A consensus clustering algorithm was then conducted to classify the patients into two clusters. Tumor prognosis, clinicopathological features, mutations, functional analysis, ferroptosis status analysis, immune infiltration, immune checkpoint-related gene expression level, chemotherapy resistance, and tumor stem cells were analyzed between clusters. An energy metabolism-related signature was further developed and verified using data from cBioPortal datasets.

**Results:** Two clusters (C1 and C2) were identified using a consensus clustering algorithm based on an energy metabolism-related signature. The patients with subtype C1 had more metabolism-related pathways, different ferroptosis status, higher cancer stem cell scores, higher chemotherapy resistance, and better prognosis. Subtype C2 was characterized by an increased number of advanced BLCA cases and immune-related pathways. Higher immune and stromal scores were also observed for the C2 subtype. A signature containing 16 energy metabolism-related genes was then identified, which can accurately predict the prognosis of patients with BLCA.

**Conclusion:** We found that the energy metabolism-associated subtypes of BLCA are closely related to the immune microenvironment, immune checkpoint-related gene expression, ferroptosis status, CSCs, chemotherapy resistance, prognosis, and progression of BLCA patients. The established energy metabolism-related gene signature was able to predict survival in patients with BLCA.

## Introduction

Bladder cancer (BC) is one of the most prevalent cancers, accounting for approximately 200,000 deaths per year worldwide, with preponderance in men compared with women (4:1) (Bray et al., 2018). More than 90% of BC cases are transitional cell carcinomas, also known as bladder urothelial carcinoma (BUC or BLCA), accounting for the majority of primary BC cases (Potts et al., 2017). BC can be divided into muscle invasive bladder cancer (MIBC) and non-muscle invasive bladder cancer (NMIBC) based on whether it invades the muscle layer of the bladder (Babjuk et al., 2017). At the time of initial diagnosis, NMIBC accounts for approximately 75% of BC cases (Hollenbeck et al., 2007). In NMIBC patients, carcinoma *in situ* (CIS), high-grade T1, and high-grade Ta tumors are considered to have a high risk of tumor recurrence and disease progression. In the clinical management of BLCA, the prognosis of tumors often depends on the histopathology and stage of cancer (Kamat et al., 2016; Babjuk et al., 2017), which provides a simple risk stratification but cannot explain the different prognoses and outcomes of patients with the same histopathology and tumor stage. Thus, it is imperative to determine new biomarkers correlated with the prognosis of patients with BLCA at an early stage.

Alterations in the energy metabolism of cancer cells compared with normal cells are an emerging hallmark of most cancers (Hanahan and Weinberg, 2011; Fumarola et al., 2018). In the different types of energy metabolism reprogramming that cancer cells may rely on, glycolysis is the most common pathway that many cancer cells may utilize, even in the presence of oxygen, to generate ATP to maintain the reduction–oxidation balance and macromolecular biosynthesis, which is required to support the growth, division, and migration of cancer cells (Vander Heiden and DeBerardinis, 2017). This phenomenon of glycolysis in the presence of oxygen is also known as the Warburg effect (Dang and Semenza, 1999). While the metabolic phenotype of some tumor cells is mainly glycolytic, some other tumors have a predominantly oxidative phosphorylation (OXPHOS) metabolic phenotype (Sonveaux et al., 2008). There is growing evidence that metabolic reprogramming of cancer cells is heterogeneous. Furthermore, it has been reported that tumor cells can also absorb free fatty acids and ketones secreted by adjacent catabolic cells, which provide energy for mitochondrial OXPHOS (Bonuccelli et al., 2010; Nieman et al., 2011). In addition, a previous study reported that glutamine-driven mitochondrial OXPHOS, rather than glycolysis, takes up most of the ATP production under hypoxic conditions (Fan et al., 2013). Concerns regarding the possibility that cancer-related energy metabolic reprogramming may provide new targeted therapies are emerging, which may have fewer side effects and higher antitumor efficiency than conventional cytotoxic chemotherapy (Tennant et al., 2010; DeBerardinis and Chandel, 2016; Luengo et al., 2017).

In this study, the energy metabolic profile and clinical value in patients with BLCA were investigated using the Cancer Genome Atlas (TCGA) and cBioPortal online sequencing data. Based on the consensus clustering analysis of the gene expression profile, patients could be classified into two robust clusters with significant differences in molecular features and tumor prognosis. Furthermore, an energy metabolism-related signature was developed to assess the prognosis of patients with BLCA in the TCGA dataset, which was then verified using data from the cBioPortal database. We found a significant association between the prognosis of patients with BLCA and the energy metabolism-related signature, which could serve as an independent clinicopathological prognostic factor. In summary, our study revealed a strong correlation between energy metabolism status and clinical prognosis of patients with BLCA.

## Methods

### Dataset Collection

Data from bladder cancer patients containing clinicopathological and transcriptomic information were downloaded from the Cancer Genome Atlas (TCGA) data portal (https://portal.gdc.cancer.gov/). Moreover, a validation cohort (*n* = 296) was derived from the cBioPortal online database (http://www.cbioportal.org/), and it validated the results (Gao et al., 2013).

### Gene Sets Containing and Consensus Clustering

The Molecular Signature Database (MSigDB, http://www.broad.mit.edu/gsea/msigdb/) was utilized to contain two energy metabolism-related gene sets (energy-requiring part of metabolism and reactome energy metabolism) (Subramanian et al., 2005; Zhou et al., 2018). After removing overlapping genes, an energy metabolism-related gene set containing 590 genes was obtained (Supplementary Datasheet S1). The “ConsensusClusterPlus” R package was then used to perform consensus clustering analysis, and the maximum number of clusters was set at 6. The “pheatmap” R package was utilized to visualize the results of the most differentially expressed energy metabolism-related genes in the form of a heatmap. Survival curves were generated using the R packages “survival.” Comparisons of clinicopathological characteristics were performed using chi-square tests or Fisher’s exact tests for categorical variables and Student’s t-tests for continuous variables.

### Differentially Expressed Gene Identification and Enrichment Analysis

The differentially expressed genes (DEGs) between groups characterized by consensus clustering were explored and visualized in volcano plots using the “ggplot” R package. The thresholds of the fold-change value and adjusted *p* value were set at 1.5 and 0.05, respectively. The top 50 upregulated and downregulated DEGs with the most differential changes are shown in the form of a heatmap. Furthermore, we used the “ClusterProfiler” R package to conduct enrichment analysis to better understand the underlying functions of the potential targets. GO functions were analyzed for the DEGs between groups identified by energy metabolism-related genes, and the KEGG pathway and gene set enrichment analysis (GSEA) were enriched. In addition, the correlation between metabolic status and CSCs was evaluated using the OCLR algorithm constructed by Malta et al. (Teo and Rosenberg, 2018).

### Immune Infiltration and Ferroptosis Status Analysis

Based on the cohorts grouped by consensus clustering, immune infiltration estimation was then conducted using the xCell and CIBERSORT algorithms in the “immunedeconv” R package and visualized in the form of a heatmap and boxplot. Furthermore, eight immune checkpoint-relevant genes (CD274, CTLA4, HAVCR2, LAG3, PDCD1, PDCD1LG2, TIGIT, and SIGLEC15) were selected and then explored in the different groups, and the “ggplot2” and “pheatmap” R packages were utilized to visualize the expression of these genes in the two groups. The Wilcox test was used for the analysis of significance between groups, and *p* < 0.05 was regarded as statistically significant. The ferroptosis status analysis was achieved through the “ggplot2” and “pheatmap” R packages.

### Gene Signature Identification

The TCGA–BLCA dataset was analyzed to determine whether the energy metabolism-related genes were correlated with the overall survival of the patients *via* univariate Cox proportional hazards regression analysis. Simultaneously, DEGs between bladder cancer and normal tissue were determined using the online tool Gene Expression Profiling Interactive Analysis 2.0. (GEPIA2; http://gepia2.cancer-pku.cn/#index) (Tang et al., 2019). A Venn diagram was constructed to select the optimal energy metabolism-related gene set with the R package “ggplot2.” Furthermore, the least absolute shrinkage and selection operator (LASSO) Cox regression algorithm was conducted through the “glmnet” R package. The nomogram and calibration curves were constructed by using the “rms” and “survival” R packages.

## Results

### Data Collection and Consensus Clustering

To explore the role of energy metabolism status in BLCA, we obtained a cohort of 408 patients using RNA sequencing data and clinicopathological information from the TCGA database. Two energy metabolism-related gene sets were then obtained, and after removing overlapping genes, an energy metabolism-related gene set containing 590 genes was obtained (Supplementary Datasheet S1).

**FIGURE 1:**
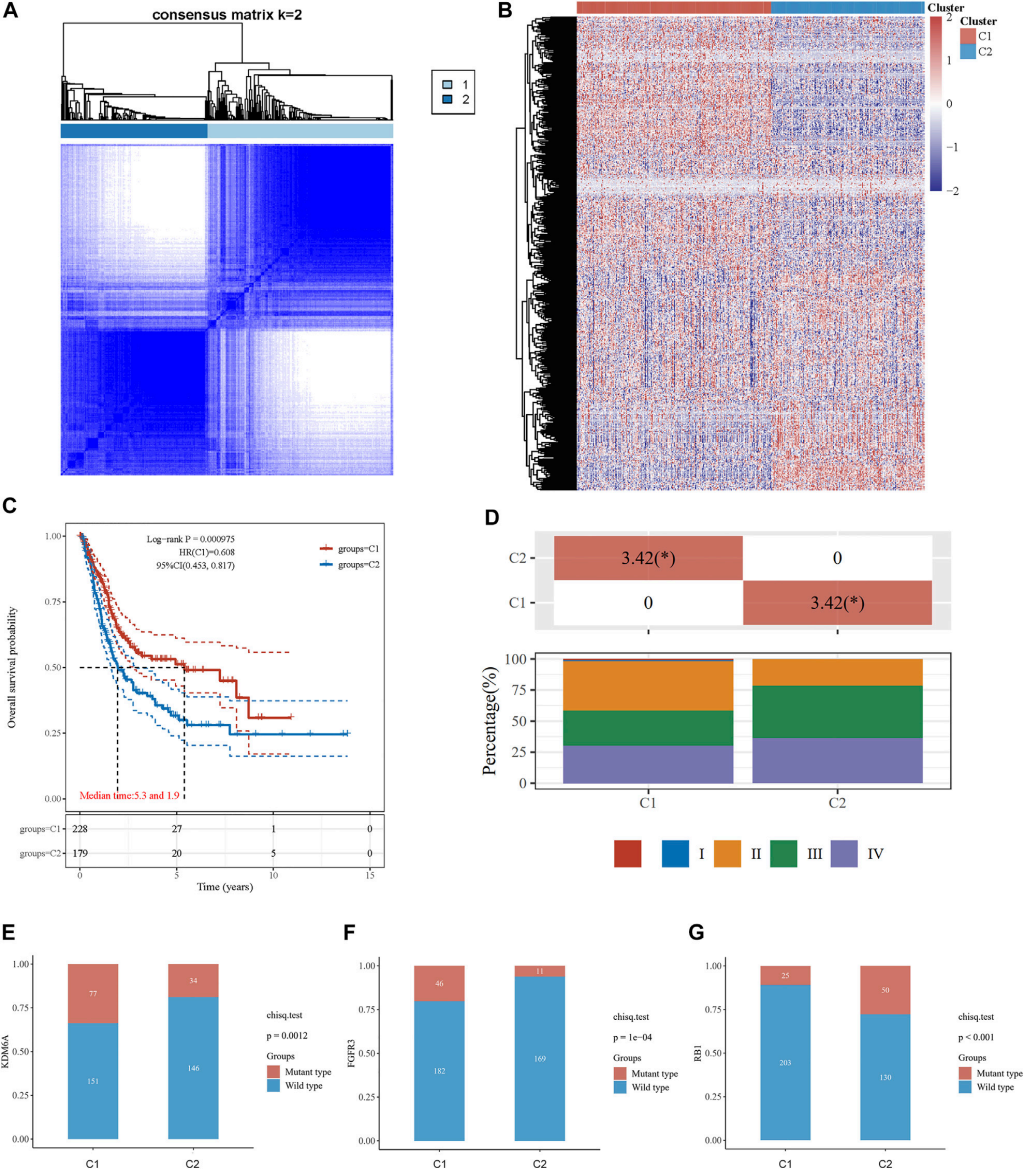
Consensus clustering analysis to identify the genomic subtype of BLCA based on an energy metabolism-related gene set. **(A)** Consensus clustering matrix of 408 samples from the TCGA dataset for k = 2. **(B)** Heatmap of energy metabolism-related gene expression in different clusters. Red represents high gene expression, and blue represents low expression. **(C)** The Kaplan–Meier curve of overall survival of BLCA patients in two clusters. **(D)** Tumor stage distribution of BLCA patients in two clusters with different metabolic statuses. The frequency of KDM6A **(E)**, FGFR3 **(F)**, and RB1 **(G)** mutations in two clusters with different metabolic statuses.

The association between energy metabolism status and prognosis of patients with BLCA was further investigated. The consensus clustering algorithm, empirical cumulative distribution function (CDF) plot, and consensus clustering matrix indicated that patients could be grouped into two groups (Figure 1A, Supplementary Figure S1). Figure 1B presents the clustering heatmap of the top variable expression genes with SD >0.1 in these two clusters grouped by the energy metabolism-related gene set. Survival curves revealed that patients in cluster 1 had a significantly longer overall survival [OS hazards ratio (HR): 0.608, 95% confidence interval (CI): 0.453–0.817, *p* < 0.001, Figure 1C] and better progression-free survival (PFS, HR: 0.691, 95% CI: 0.513–0.931, *p* = 0.0151, Supplementary Figure S1) than those in cluster 2.

The clinicopathological features of the two clusters were explored to investigate the differences between the clusters. Survival status and race were significantly different between the two clusters (Table 1). In addition, patients in cluster 1 tended to have no metastasis, relatively earlier tumor stage (Figure 1D), and relatively lower histologic grade, whereas more metastasis, higher tumor stage, and higher tumor grade were observed in patients from cluster 2. The top 20 most frequently mutated genes in each cluster were then compared, and we found that the frequency of KDM6A (*p* = 0.001, Figure 1E) and FGFR3 (*p* < 0.001, Figure 1F) mutations were higher in C1, whereas the frequency of RB1 mutations (*p* < 0.001, Figure 1G) in C2 was higher.

**TABLE 1:** Clinicopathological feathers between the two clusters identified by energy metabolic-related gene set.

Characteristics	C1 (*n* = 228)	C2 (*n* = 180)	*p* Value
Survive			<0.001
** **Alive	148 (64.9%)	81 (45%)	
** **Dead	80 (35.1%)	99 (55%)	
Gender			0.098
** **Female	52 (22.8%)	55 (30.6%)	
** **Male	176 (77.2%)	125 (69.4%)	
Race			<0.001
** **Asian	38 (16.7%)	6 (3.3%)	
** **Black	13 (5.7%)	10 (5.6%)	
** **White	166 (72.8%)	158 (87.8%)	
Metastasis			0.001
** **M0	129 (56.6%)	67 (37.2%)	
** **M1	6 (2.6%)	5 (2.8%)	
** **MX	92 (40.4%)	106 (58.9%)	
Stage			<0.001
** **I	2 (0.9%)	0	
** **II	91 (39.9%)	39 (21.7%)	
** **III	64 (28.1%)	76 (42.2%)	
** **IV	69 (30.3%)	65 (36.1%)	
Histologic grade			<0.001
** **High	205 (89.9%)	179 (99.4%)	
** **Low	21 (9.2%)	0	

### Enrichment Analysis

**FIGURE 2:**
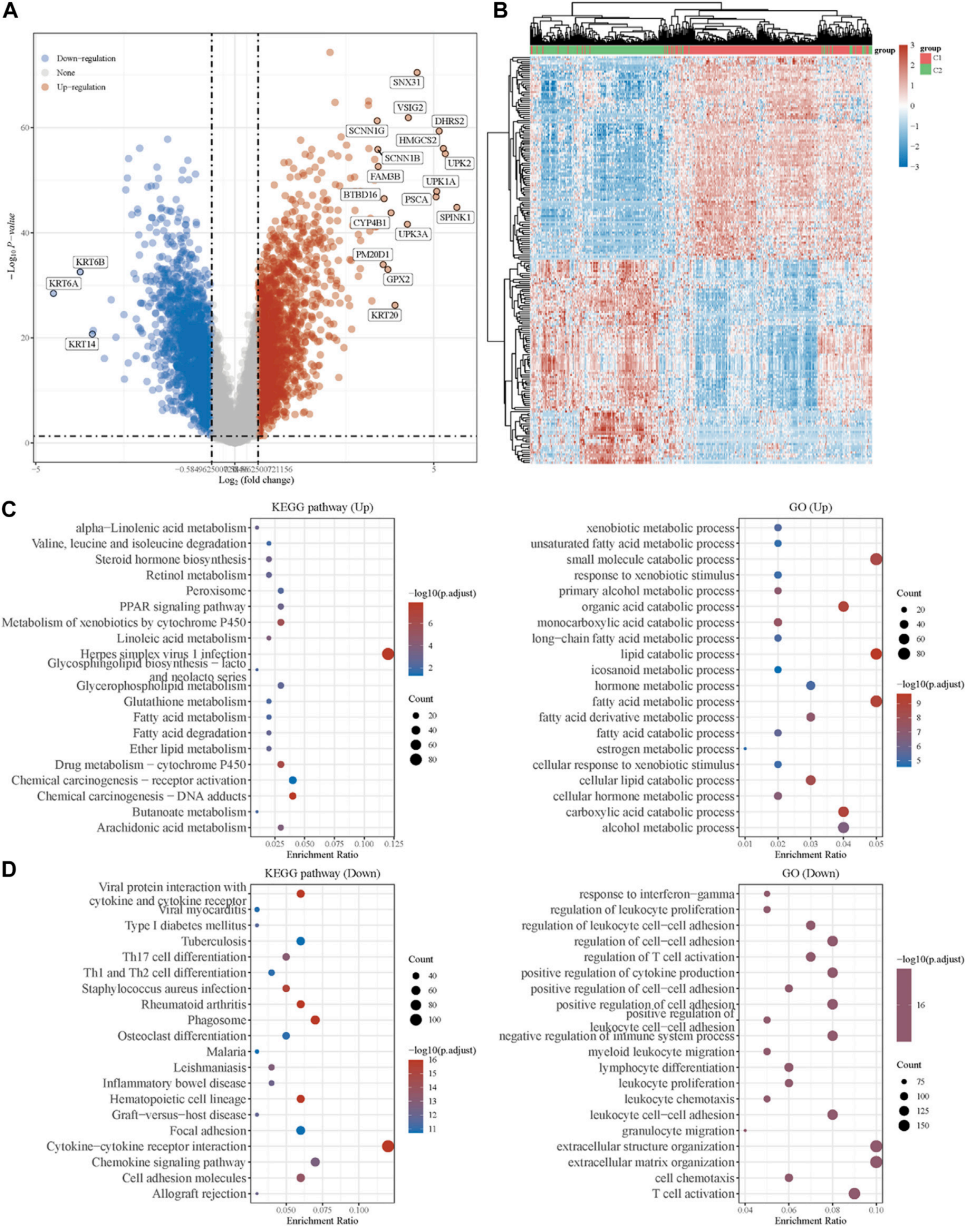
Identification of DEGs between the two clusters grouped by energy metabolism-related gene set and functional enrichment analysis. **(A)** Volcano plot of DEGs between two clusters with different metabolic statuses. The red and blue points represent up- and downregulated genes with statistical significance, respectively. **(B)** Heatmap of the top 50 up- and downregulated genes with the most differential changes. **(C)** GO/KEGG analysis of DEGs that were upregulated in cluster 1. **(D)** GO/KEGG analysis of DEGs that were downregulated in cluster 1. DEG: differentially expressed genes.

To explore the underlying mechanism of the difference between the two clusters, DEGs between the two clusters were identified. As shown in Figure 2A, the volcano plot indicated the upregulated genes (SNX31, VSIG2, DHRS2, HMGCS2, *etc*.) and downregulated genes (KRT6B, KRT6A, KRT14, *etc*.) in cluster 1 as compared to cluster 2. The top 50 upregulated and downregulated genes were then displayed in the form of a heatmap (Figure 2B).

**FIGURE 3:**
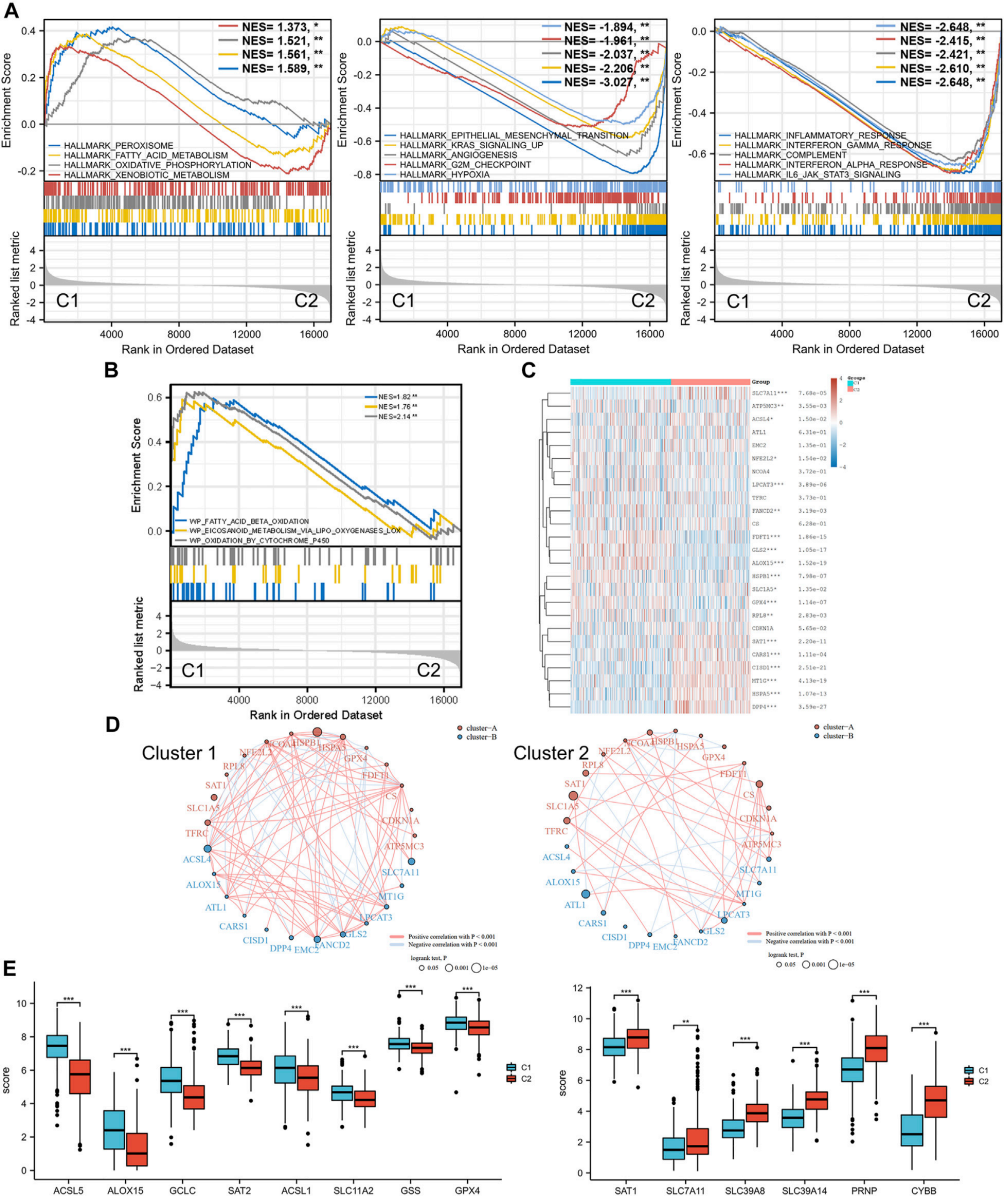
Ferroptosis status analysis and GSEA results. **(A)** GSEA of DEGs that were upregulated in cluster 1 and cluster 2. **(B)** GSEA results of lipid oxidation metabolism terms. **(C)** Heatmap of ferroptosis-related gene expression in two different metabolic status clusters. **(D)** Ferroptosis-related gene interaction network. The red and blue lines represent the positive and negative correlation, respectively. The thickness of the line represents the correlation between the two genes. The larger circle indicates a more significant prognostic log rank *p* value. **(E)** Expression difference of ferroptosis-related genes between the two clusters. GSEA: gene set enrichment analysis. DEG: differentially expressed genes. *p < 0.05, **p < 0.01, ***p < 0.001.

Moreover, with the thresholds of the fold-change value and adjusted *p* value setting at 1.5 and 0.05, up- and downregulated genes were selected for functional enrichment analysis. KEGG analysis of the most relevant signaling pathways in cluster 1 was mainly associated with the energy metabolism (Figure 2C). The results of GO analysis showed the same trend, and the most enriched terms in the biological process (BP), molAUCecular function (MF), and cellular component (CC) were strongly correlated with the energy metabolism (Figure 2C), mainly enriched in small-molecule catabolic processes, lipid catabolic processes, and fatty acid metabolic processes. However, the GO/KEGG analysis of the downregulated genes in cluster 1, indicating that they were upregulated in cluster 2, showed different results. GO analysis of cluster 2 was mainly enriched in immune terms, including T-cell activation, leukocyte cell–cell adhesion, negative regulation of immune system process, regulation of leukocyte cell–cell adhesion, and regulation of T-cell activation. In the KEGG pathways, the results showed that the DEGs were mainly enriched in cytokine–cytokine receptor interactions, chemokine signaling pathways, and immune-related pathways, including those involving phagosomes, Th17 cell differentiation, and Th1 and Th2 cell differentiation. GSEA results of cluster 1 also showed metabolism-associated terms, while cluster 2 was mainly enriched in immune terms (Figure 3A, Supplementary Figure S2). These results indicated that cluster 1 could be characterized by the activation of oncometabolic processes, while cluster 2 may be characterized by the upregulation of tumor-related immunogenicity.

### Ferroptosis Status Analysis

Ferroptosis, driven by excessive lipid peroxidation, is an iron-dependent regulated cell death that is related to the development and treatment response of various types of tumors (Chen et al., 2021). The enrichment results of the GSEA indicated that the two clusters were different in terms of fatty acid beta oxidation, eicosanoid metabolism *via* lipoxygenases lox, and oxidation by cytochrome p450 (Figure 3B). These lipid oxidation metabolism characteristics, which were correlated to ferroptosis, suggested that the ferroptosis status of the two clusters may differ. Analysis of ferroptosis-related genes between the two groups revealed that many ferroptosis-related genes were differentially expressed between the two clusters (Figures 3C,E), and the expression levels of some genes (including ACSL5, ACSL1, GSS, SLC7A11, SCL39A8, SLC39A14, and PRNP) were significantly associated with the prognosis of patients with BLCA (Supplementary Figure S3). In addition, the correlation between the ferroptosis-related genes was more obvious in cluster 1 and was dominated by a positive correlation, while the correlation between different genes in the ferroptosis-related gene network of C2 was weaker (Figure 3D). SLC7A11 was significantly downregulated in cluster 1, and the prognostic effect of SLC7A11 in cluster 1 was more significant than that in cluster 2. Similarly, SAT1 was significantly upregulated in cluster 2, and the prognostic effect in cluster 2 was more significant. This finding suggested that the metabolism status of BLCA patients was significantly associated with the expression of selected ferroptosis-related genes, some of which were correlated with the prognosis of BLCA.

### Immune Infiltration Analysis

**FIGURE 4:**
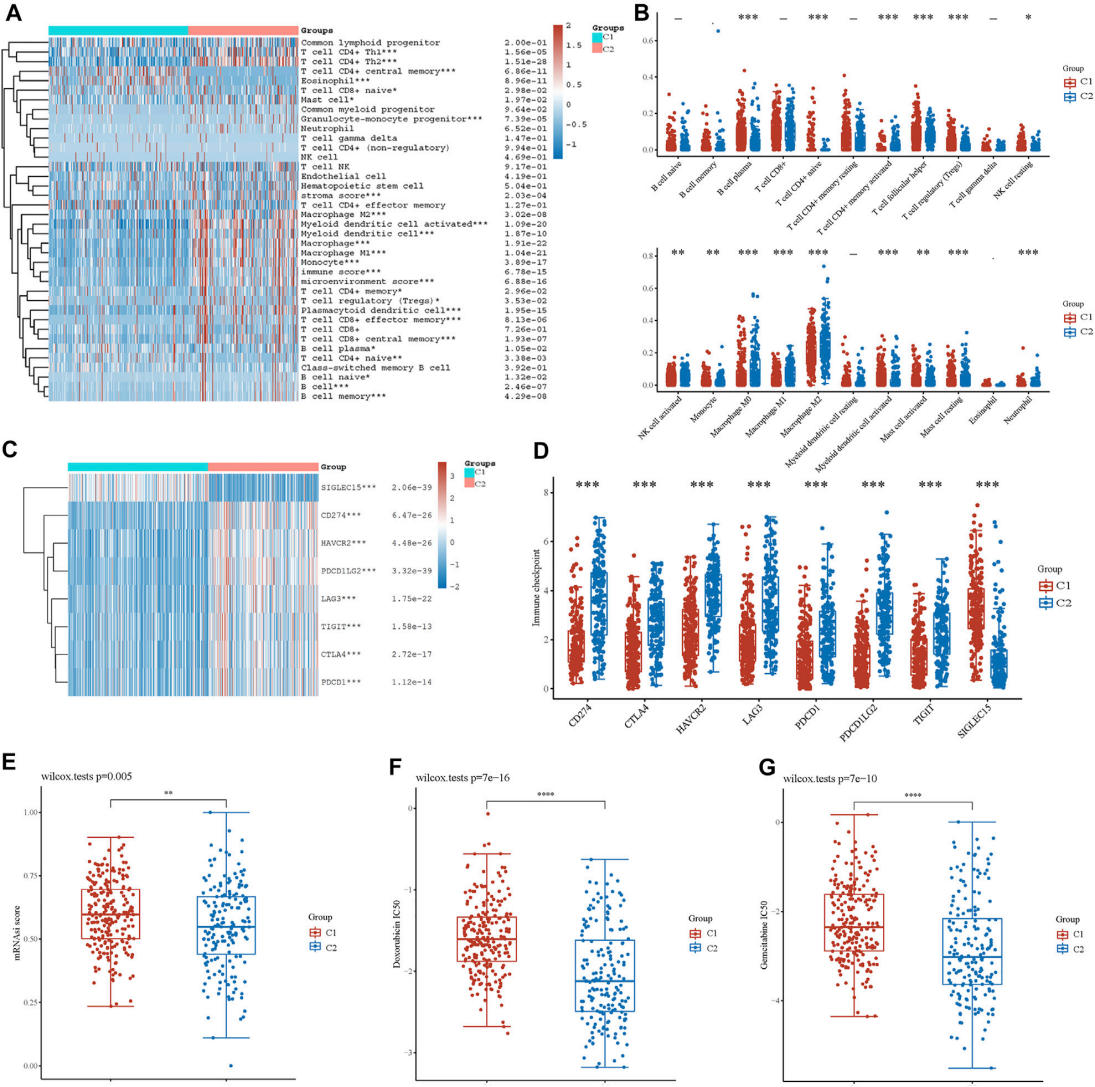
Analysis of immune cell infiltration between two clusters. **(A)** Immune cell score heatmap by xCell algorithm. Red represents high expression/score, whereas blue represents low expression/score. **(B)** Boxplot of immune infiltration status in two clusters by the CIBERSORT algorithm. Heatmap **(C)** and boxplot **(D)** of immune checkpoint-related gene expression between two clusters. Cancer stem cell scores **(E)** and chemotherapy resistance to doxorubicin **(F)** and gemcitabine **(G)** of the two clusters. *p < 0.05, **p < 0.01, ***p < 0.001.

Based on the enrichment analysis results that upregulated genes in cluster 2 were correlated with tumor immune function in BLCA, the immune infiltration status of the two clusters was then examined. A heatmap of immune cell infiltration suggested that the tumor immune microenvironment was significantly different between the two clusters (Figure 4A). Cluster 2 had higher infiltration levels of T-cell CD4^+^ Th1, T-cell CD4^+^ Th2, T-cell CD4^+^ memory, T-cell regulatory, T-cell CD4^+^ naïve, granulocyte-monocyte progenitor, macrophage, M1 macrophage, M2 macrophage, myeloid dendritic cell, activated myeloid dendritic cells, monocytes, mast cells, plasmacytoid dendritic cells, T-cell CD8^+^, T-cell CD8^+^ effector memory, T-cell CD8^+^ central memory, B-cell plasma, B-cell naïve, B cell, and B-cell memory. In addition, high infiltration levels of CD4^+^ central memory T cells, eosinophils, and CD8^+^ naïve T cells were observed in cluster 2. Boxplots showed similar results using the CIBERSORT algorithm (Figure 4B). The Spearman correlation analysis also revealed a significant association between energy metabolism-related gene set risk scores and the infiltration of CD4^+^ T cells, CD8^+^ T cells, neutrophils, macrophages, and myeloid dendritic cells (Supplementary Figure S4). This result is consistent with the conclusion of the enrichment analysis, indicating that patients with BLCA from cluster 2 had higher immune cell infiltration and that the energy metabolism-related gene set had a potential correlation with the tumor immune microenvironment.

Furthermore, we investigated the expression of immune checkpoint (IC)-related genes between the two clusters. In cluster 2, we found a relatively higher expression of IC-related genes, including CD274, CTLA4, HAVCR2, LAG3, PDCD1, PDCD1LG2, and TIGIT (Figures 4C,D). However, SIGLEC15 expression was higher in cluster 1. Thus, energy metabolism-related genes were significantly correlated with biomarkers of immune checkpoints and may play an important role in immunological therapy for BLCA.

### Cancer Stem Cells and Drug Sensitivity Analysis

Based on the gene expression profile containing 11,774 CSC-associated genes, analysis of CSCs was conducted, and patients in cluster 1 had a higher CSC score, indicating a significant correlation between energy metabolic status and CSCs (Figure 4E). Next, drug sensitivity was evaluated between the two clusters. Figures 4F,G show that energy metabolic status was significantly associated with the IC50 scores of doxorubicin and gemcitabine for BLCA.

### Energy Metabolism-Related Gene Signature Identification

**FIGURE 5:**
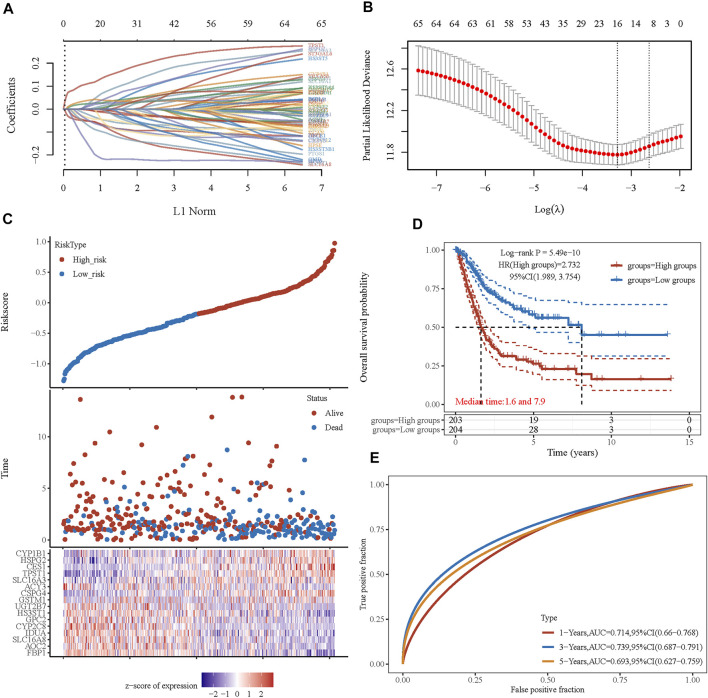
Prognostic signature was established based on four prognostic energy metabolism-related genes. **(A)** LASSO coefficient profiles of the genes associated with the metabolism of BLCA. **(B)** Partial likelihood deviance is plotted versus log(λ). **(C)** The risk score of each sample based on the energy metabolism-related gene set. Patients were divided into low-risk and high-risk groups according to the median value of the risk score. The high/low expression levels of 16 genes which were involved in the prognostic signature are shown in red/blue in each sample. **(D)** The Kaplan–Meier curve of overall survival differences stratified by signature risk score. **(E)** The ROC curves of the signature for overall survival at 1, 3, and 5 years. LASSO: least absolute shrinkage and selection operator. ROC: receiver operating characteristic.

Considering the close correlation between the prognosis of patients with BLCA and energy metabolism status, we suggest developing an energy metabolism-related gene signature for prognosis prediction. Based on DEGs between cluster 1 and cluster 2, Venn diagrams were constructed and showed that 162 of 590 metabolism-related genes were differentially expressed between clusters (Supplementary Figure S5). Then, 67 of 162 metabolism-related genes were further identified with a significant relevance to the OS of patients with BLCA (*p* < 0.1). To ensure the feasibility and stability of the clinical prognostic value of these 67 genes, LASSO analysis was conducted, and we obtained 16 energy–metabolism-correlated genes associated with the prognosis of patients with BLCA, including FBP1, AOC2, SLC16A8, IDUA, CYP2C8, GPC2, HS3ST1, UGT2B7, GSTM1, CSPG4, ACY3, SLC16A3, TPST1, CES1, HSPG2, and CYP1B1 (Figures 5A,B). Therefore, based on the Cox coefficient, the energy metabolism-related gene-based prognostic signature (EMRGPS) was calculated as follows: risk score= (−0.055 × FBP1 expression) + (−0.0085 × AOC2 expression) + (−0.0567 × SLC16A8 expression) + (−0.0351 × IDUA expression) + (−0.0444 × CYP2C8 expression) + (−0.0627 × GPC2 expression) + (−0.0885 × HS3ST1 expression) + (−0.0096 × UGT2B7 expression) + (0.0197 × GSTM1 expression) + (0.026 × CSPG4 expression) + (−0.1969 × ACY3 expression) + (0.0445 × SLC16A3 expression) + (0.117 × TPST1 expression) + (0.0421 × CES1 expression) + (0.0264 × HSPG2 expression) + (0.0051 × CYP1B1 expression).

**FIGURE 6:**
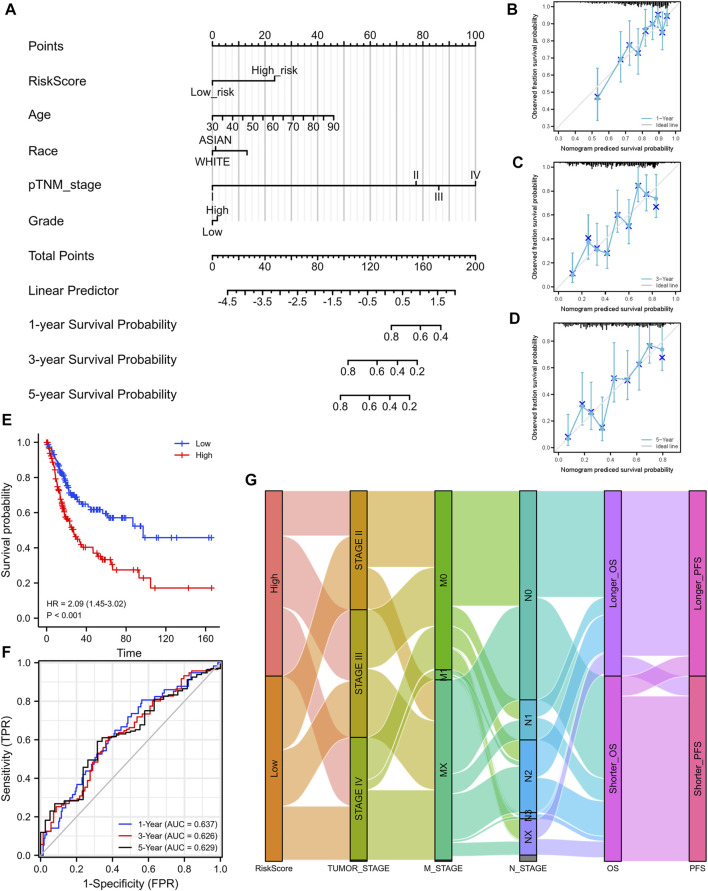
Construction of a nomogram and the independent signature validation. **(A)** Nomogram for predicting 1-, 3-, or 5‐year OS in patients with BLCA. **(B)** The calibration plots for predicting 1-year OS. **(C)** The calibration plots for predicting 3-year OS. **(D)** The calibration plots for predicting 5-year OS. **(E)** Validation of the signature in overall survival based on data from the cBioPortal online database. **(F)** The ROC curves of the signature validation for overall survival at 1, 3, and 5 years. **(G)** Sankey diagram showing the association between signature risk scores and clinicopathological characteristics based on data from the cBioPortal database. OS: overall survival. ROC: receiver operating characteristic.

Based on the median value of the risk score, patients with BLCA could be categorized into low-risk and high-risk groups (Figure 5C). The Kaplan–Meier curve indicated that patients in the high-risk group had a significantly poorer OS than those in the low-risk group (Figure 5D, *p* < 0.001), and the AUCs for 1-, 3-, and 5-year OS were 0.714, 0.739, and 0.693, respectively (Figure 5E). Furthermore, to ensure the prediction value of EMRGPS, an independent cohort from the cBioPortal online database served as a validation set to verify our results. Survival curves showed similar results, and significantly worse OS was observed in the low-risk group than in the high-risk group in patients from the cBioPortal online database (Figure 6E). The AUCs for 1-, 3-, and 5-year OS in the validation cohort were 0.637, 0.626, and 0.629, respectively (Figure 6F). The association between signature risk scores and clinicopathological characteristics of the validation cohort was presented in the form of a Sankey diagram (Figure 6G).

**FIGURE 7:**
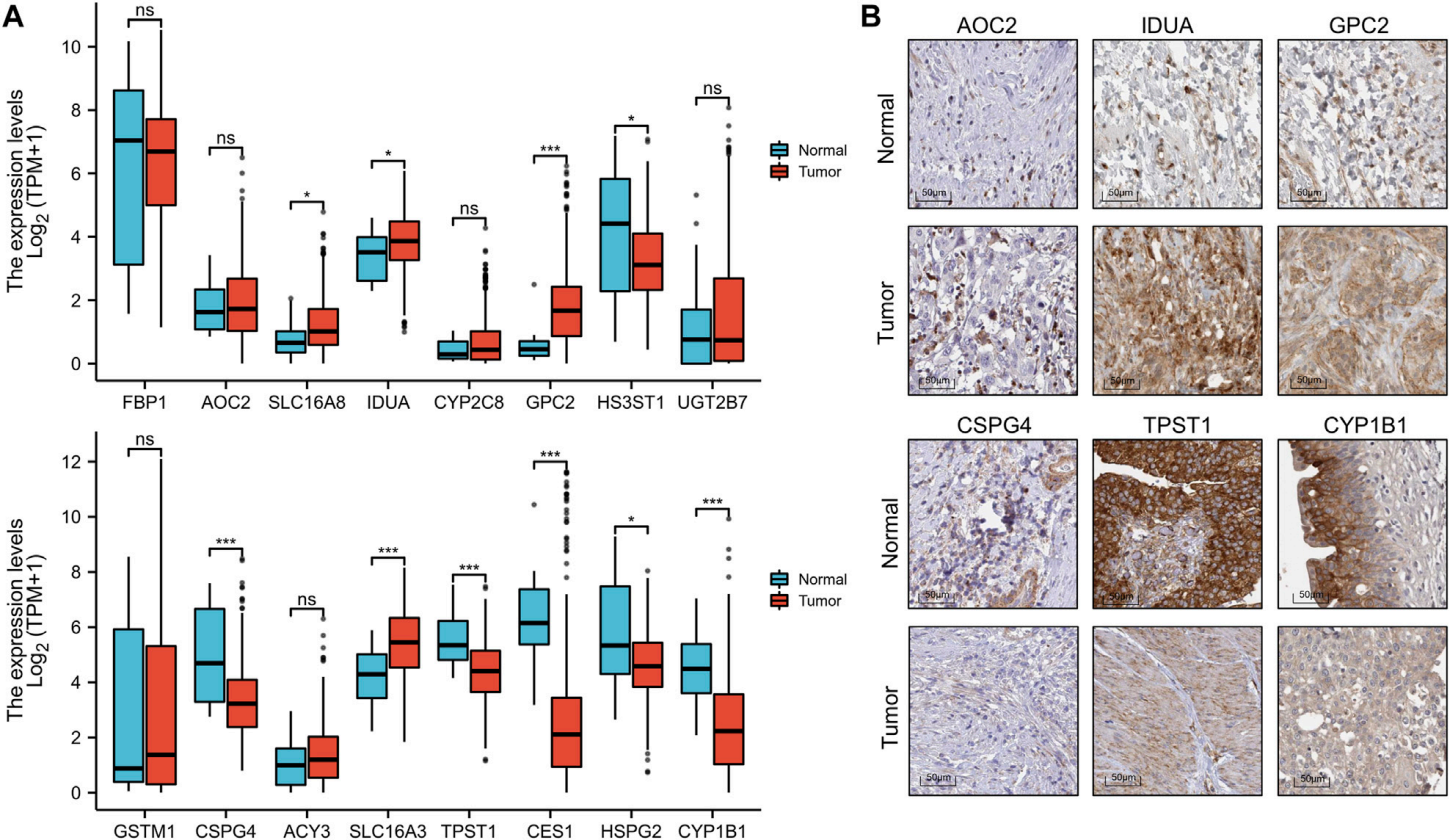
Box plot of the expression difference of 16 genes involved in the prognostic signature between normal and BLCA tissue **(A)**. The expression difference of AOC2, IDUA, GPC2, CSPG4, TPST1, and CYP1B1 between tumor and normal tissue at the protein level according to the Human Protein Atlas (HPA) cohort. **(B)**. *p < 0.05, **p < 0.01, ***p < 0.001.

To better predict the prognostic value of EMRGPS in patients with BLCA, a nomogram using available clinicopathological parameters and the risk score of the signature was constructed (Figure 6A). Moreover, calibration curves using 1-, 3-, and 5-year survival rates were developed to estimate the accuracy of the nomogram (Figures 6B–D). The multivariate and univariate Cox regression analyses of EMRGPS and other clinicopathological characteristics for OS are presented in Table 2. The signature risk score was an independent factor for the prognosis of patients with BLCA (HR: 2.443, 95% CI: 1.758–3.395, *p* < 0.001). Furthermore, the survival analysis (Supplementary Figure S6) and the different expression patterns (Figure 7A) of the 16 genes involved in EMRGPS between normal and tumor tissues were explored in the TCGA cohort (Figure 7). Of the 16 metabolism-related genes, we found that AOC2, IDUA, GPC2, CSPG4, TPST1, and CYP1B1 were differentially expressed between tumor and normal tissue at the protein level according to the Human Protein Atlas (HPA) cohort (Figure 7B).

**TABLE 2:** Multivariate and univariate Cox regression analyses of EMRGPS and other clinicopathologic characteristics for OS in the TCGA cohort.

Overall survival	Univariate analysis			Multivariate analysis		
	HR	95% CI	*p* Value	HR	95% CI	*p* Value
TCGA cohort						
Age	1.033	1.017–1.049	<0.001	1.029	1.013–1.045	<0.001
Gender (female vs. male)	1.196	0.856–1.670	0.295			
Race			0.264			
White	Reference					
Asian	0.624	0.318–1.227	0.171			
Black	1.258	0.713–2.220	0.427			
Tumor stage (III–IV vs. I–II)	2.123	1.463–3.081	<0.001	1.819	1.250–2.646	0.002
Grade (low vs. high)	0.346	0.085–1.397	0.136			
Risk Score (high vs. low)	2.712	1.959–3.757	<0.001	2.443	1.758–3.395	<0.001

Feathers with *p* value <0.1 were involved in multivariate Cox regression analyses. EMRGPS: energy metabolism-related gene-based prognostic signature.

## Discussion

The phenomenon of cancer cells shifting their metabolic pathways from oxidative phosphorylation to glycolysis for the production of sufficient adenosine triphosphate (ATP) and necessary macromolecular biosynthesis, also known as the Warburg effect, was first described in the 1920s (Weinhouse, 1956). Since its initial establishment, much effort has been made to better understand the potential mechanisms of cancer metabolic reprogramming. Growing evidence has shown that agents targeting cellular energetics in multiple pathways are involved in alterations in cancer metabolism (Jain et al., 2012). In this study, the association between energy metabolic status and the prognosis of patients with BLCA was evaluated based on RNA sequencing data from TCGA and cBioPortal online databases. A significant correlation between clinicopathological features and energy metabolism was observed, indicating that energy metabolism and BLCA are closely linked.

Functional enrichment analysis revealed a strong association between energy metabolic status and immune and inflammatory responses, suggesting an interface between energy metabolic status and the tumor immune microenvironment. Several recent studies have reported numerous alterations in the metabolic status of bladder cancer, indicating that tumor metabolic status may play a role in the tumor immune microenvironment (Woolbright et al., 2018). It was found that the immune system could be affected by lactic acid accumulated from the aerobic glycolysis process of tumor cells, which includes the enhancement of cytokine transcription and inhibition of the differentiation of monocytes into dendritic cells (Becker et al., 2013; Ghesquière et al., 2014). Oresta et al. found that mitochondrial metabolism is reprogrammed to control the induction of immunogenic cell death and the efficacy of chemotherapy for bladder cancer by increasing OXPHOS (Oresta et al., 2021). Our study showed a similar result, in which a significant correlation between metabolic status and resistance to chemotherapy, including doxorubicin and gemcitabine, was observed. Wang et al. reported that the inhibition of pyruvate kinase M2, a glycolytic enzyme for the Warburg effect, could significantly reduce chemoresistance to cisplatin in bladder cancer (Wang et al., 2017). We also found that energy metabolism was significantly correlated with most ICI biomarkers, which acted as biomarkers and immune checkpoint inhibitors or participated in the tumorigenesis and progression of BLCA. Checkpoint inhibitors have recently been approved as second-line treatments, which may alter the pattern of bladder cancer treatment (Teo and Rosenberg, 2018). This result indicates that energy metabolic status may affect the tumor microenvironment through immune cell infiltration and therefore mediate carcinogenesis of BLCA, and may play an important role in the sensitivity and resistance of immune therapy. A recent study found that mutations in peroxisome proliferator-activated receptor gamma (PPARγ), a transcription factor connecting glucose and fatty metabolism, led to immune suppression, such as inhibiting the infiltration of CD8^+^ T cells in the tumor microenvironment, which may play an important role in checkpoint inhibition in BLCA (Korpal et al., 2017).

Using the OCLR algorithm constructed by Malta et al. (Malta et al., 2018), we found that BLCA metabolic status was significantly correlated with CSCs. CSCs are a population of undifferentiated cells exhibiting stem-like features, with high tumorigenic capacity to recreate the heterogeneity of the primary tumor and serve as a major culprit for recurrence in bladder cancer (Chan et al., 2010; van der Horst et al., 2012). Previous studies have reported that CSCs are resistant to conventional therapies, including chemotherapy, radiation, and immunotherapy (Bao et al., 2006; Li et al., 2008; Radvanyi, 2013). The potential mechanism of CSCs with energy metabolic status in patients with BLCA requires further investigation.

Due to the strong association between energy metabolic status and clinicopathological characteristics in patients with BLCA, a signature was established to stratify patients into high- or low-risk of poor prognosis. Von Rundstedt et al. reported a 30-gene metabolic signature that was significant in predicting survival in patients with BLCA (von Rundstedt et al., 2016). In this study, with the application of a combination of lasso regression, a signature of 16 genes showed a powerful effect on survival prediction. Of the 16 metabolism-related genes, we found AOC2, IDUA, GPC2, CSPG4, TPST1, and CYP1B1 to be differentially expressed between tumor and normal tissues at the protein level. The AOC2 gene encodes retina-specific amine oxidase, which oxidizes aromatic monoamines such as p-tyramine, tryptamine, and 2-phenylethylamine. Its physiological role is still unclear, but a previous study suggested that AOC2 plays a role in hereditary retinal diseases (Lopes de Carvalho et al., 2019). IDUA encodes an enzyme that is correlated to the degradation of two glycosaminoglycans, and mutations in this gene lead to the autosomal recessive disease mucopolysaccharidosis type I (Ghosh et al., 2017). GPC2 belongs to a six-member human glypican family of proteins and is highly expressed in neuroblastoma (Li et al., 2017). CSPG4 represents an integral membrane chondroitin sulfate proteoglycan, which is highly expressed in human malignant melanoma cells (Uranowska et al., 2021). TPST1 encodes an integral membrane glycoprotein of the trans-Golgi network, catalyzing the tyrosine O-sulfation of soluble and membrane proteins that pass through this compartment. TPST1 encodes an integral membrane glycoprotein of the trans-Golgi network, catalyzing the tyrosine O-sulfation of soluble and membrane proteins that pass through this compartment. A previous study reported that TPST1 is highly expressed in breast carcinoma, oral squamous cell carcinoma, and soft tissue sarcoma (Jiang et al., 2015). This gene encodes a member of the cytochrome P450 superfamily of enzymes. Cytochrome P450 proteins are monooxygenases that catalyze many reactions involved in drug metabolism and synthesis of cholesterol, steroids, and other lipids (Dong et al., 2021).

The main limitation of this study is that most analyses were conducted at the mRNA level; further analysis at the protein level is imperative. Moreover, our results were mainly based on the TCGA and cBioPortal datasets. Although the large number of cases from these databases may decrease the risk of bias, another independent cohort is needed to validate our results and minimize the bias.

In conclusion, we found that energy metabolic status is closely related to the immune microenvironment, IC-related genes, CSCs, chemotherapy resistance, prognosis, and recurrence in patients with BLCA. The energy metabolism-related gene signature was then developed to predict the survival of patients with BLCA. In the era of precision medicine, this signature could provide an effective tool to meet the clinical requirements of BLCA management to some extent.

## Data Availability

The original contributions presented in the study are included in the article/Supplementary Material, further inquiries can be directed to the corresponding authors.

## Supplementary Material

Supplementary Datasheet S1 An energy metabolism-related gene set containing 590 genes.

Supplementary Figure S1 Consensus clustering matrix of 408 samples from TCGA dataset for k =3 **(A)**, 4 **(B)**, 5 **(C)**, and 6 **(D)**. Kaplan–Meier curve of progression-free survival of patients with BLCA in two clusters **(E)**. Cumulative distribution function (CDF) plot of consensus clustering for k = 2 to k = 6 **(F)**.

Supplementary Figure S2 GSEA of cluster-correlated gene sets with the top normalized enrichment score (NES) score through Joy Plot.

Supplementary Figure S3 Survival curves of ferroptosis-related genes on overall survival in patients with BLCA.

Supplementary Figure S4 Spearman correlation analysis of the energy metabolism-related gene risk score and immune infiltration level, including B cells **(A)**, CD4+ T cells **(B)**, CD8+ T cells **(C)**, neutrophils **(D)**, macrophages **(E)**, and myeloid dendritic cells **(F)**.

Supplementary Figure S5 Venn diagram identifies 162 prognostic energy metabolism-related genes that were differentially expressed between cluster 1 and cluster 2 **(A)**. Col1: 3568 genes that were differentially expressed between clusters Col2: 590 metabolism-related genes. Venn diagram identifies 67 differentially expressed energy metabolism-related genes that are significantly correlated with overall survival **(B)**. Col1: 162 metabolism-related genes that were differentially expressed between clusters 1 and 2. Col2: 157 metabolism-related genes were correlated with overall survival (*p* < 0.1).

Supplementary Figure S6 Survival curves of 16 genes involved in the prognostic signature.
