# Supplementary figures and images for: Integrated Analysis of Energy Metabolism Signature-Identified Distinct Subtypes of Bladder Urothelial Carcinoma
Source: Front Cell Dev Biol. 2022 Feb 23;10:814735. doi: 10.3389/fcell.2022.814735 (PMC8905247; doi:10.3389/fcell.2022.814735)

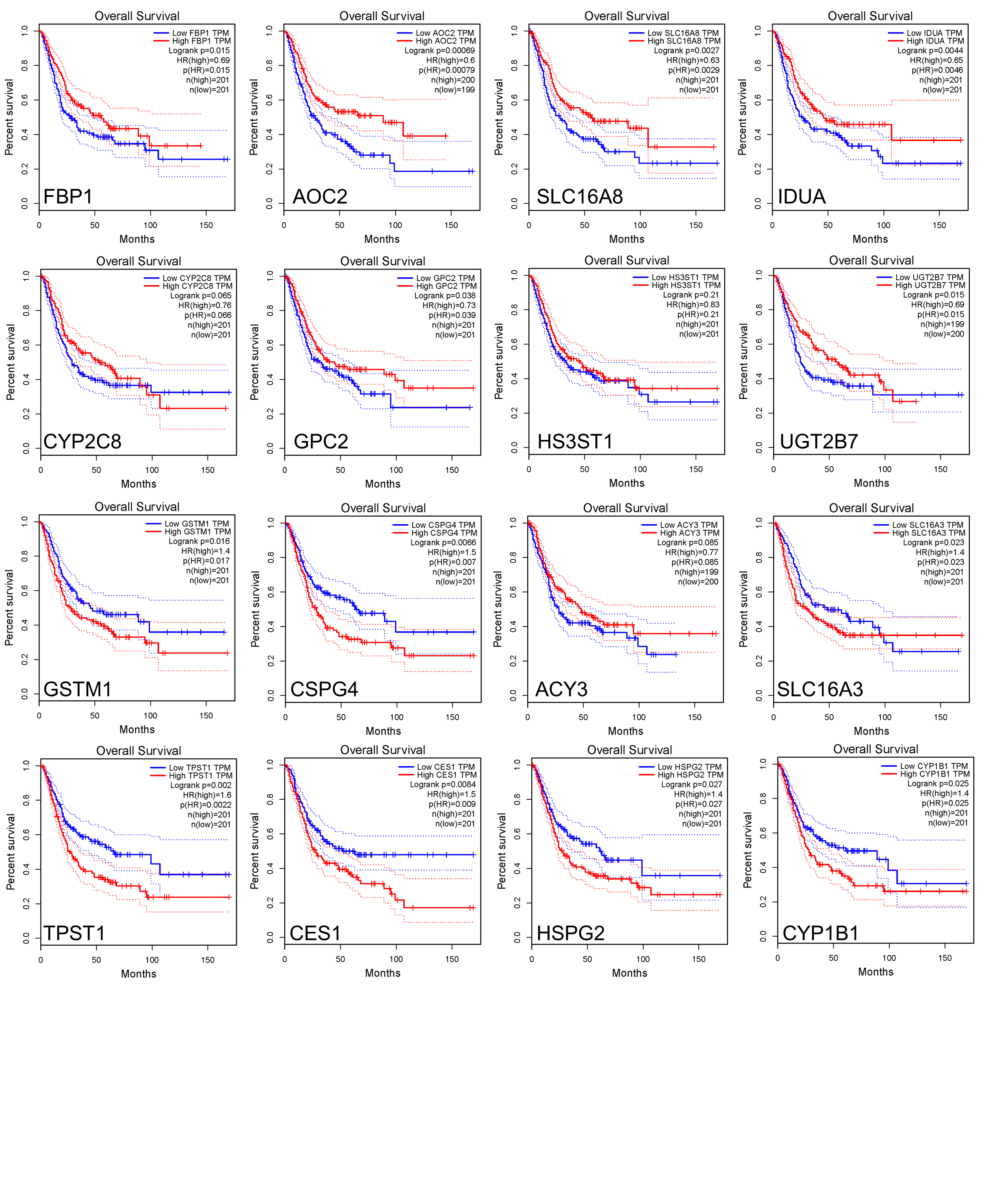

Supplement: Supplementary file 1 [file Image6.TIF]

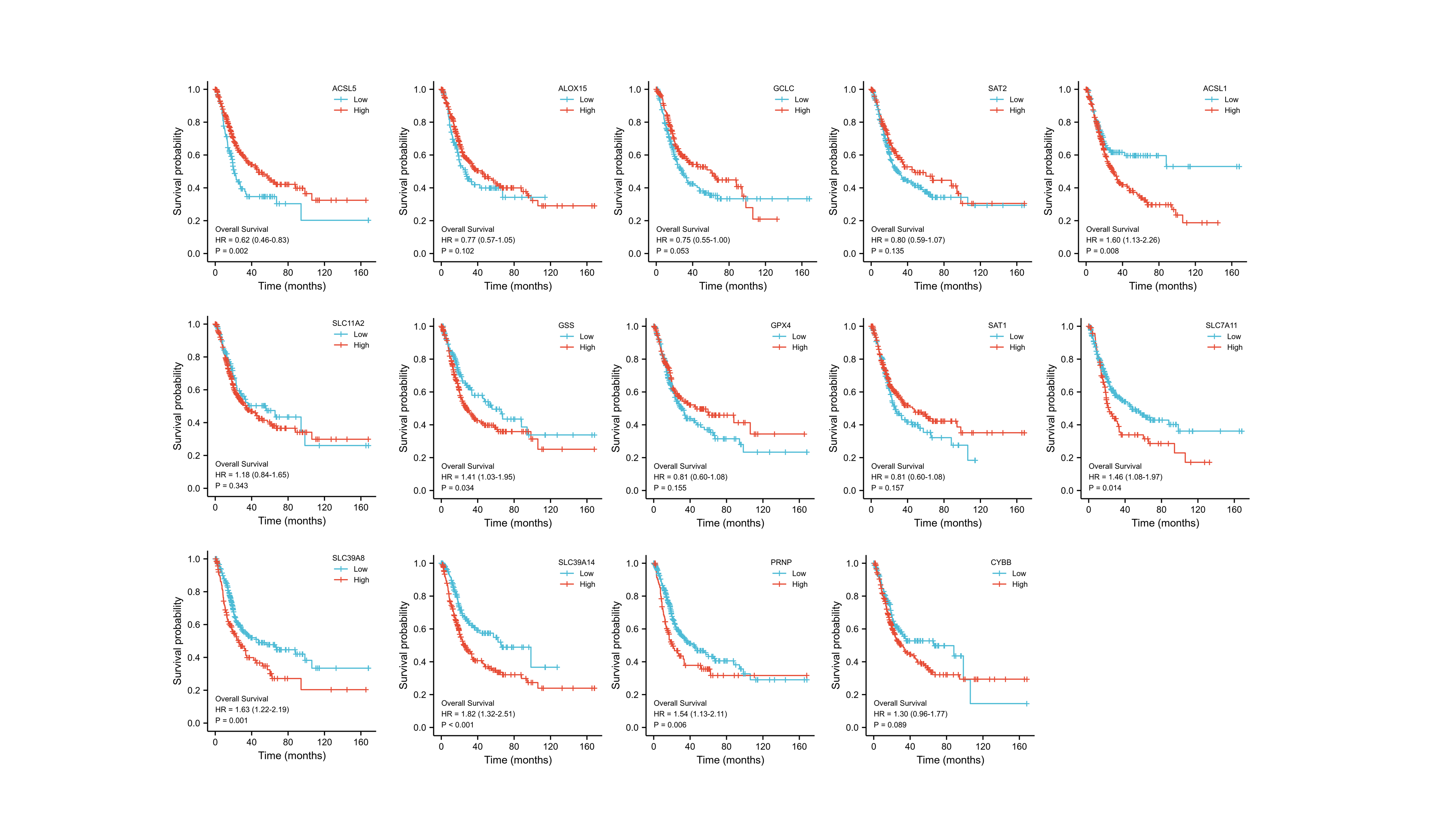

Supplement: Supplementary file 2 [file Image3.TIF]

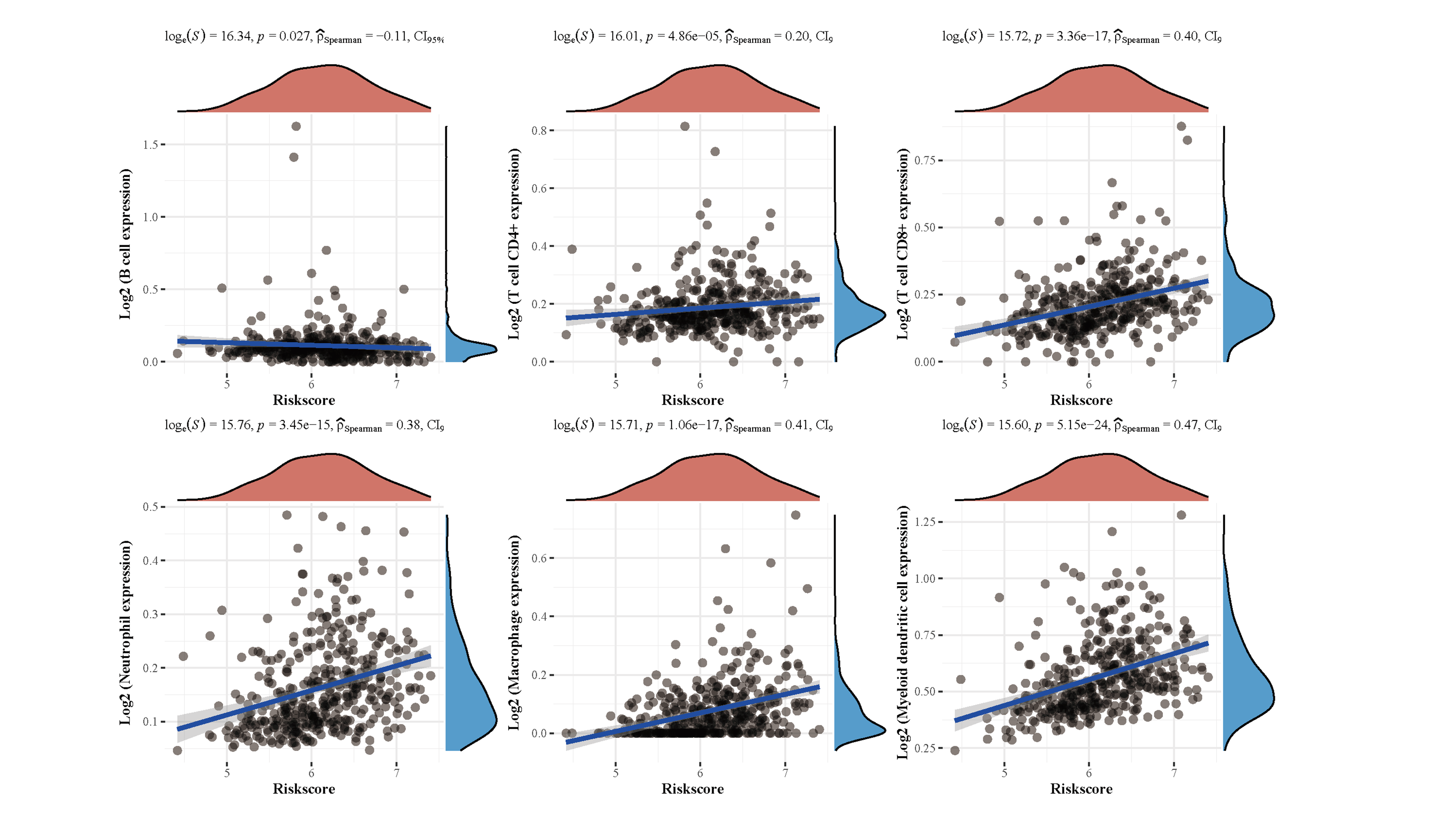

Supplement: Supplementary file 3 [file Image4.TIF]

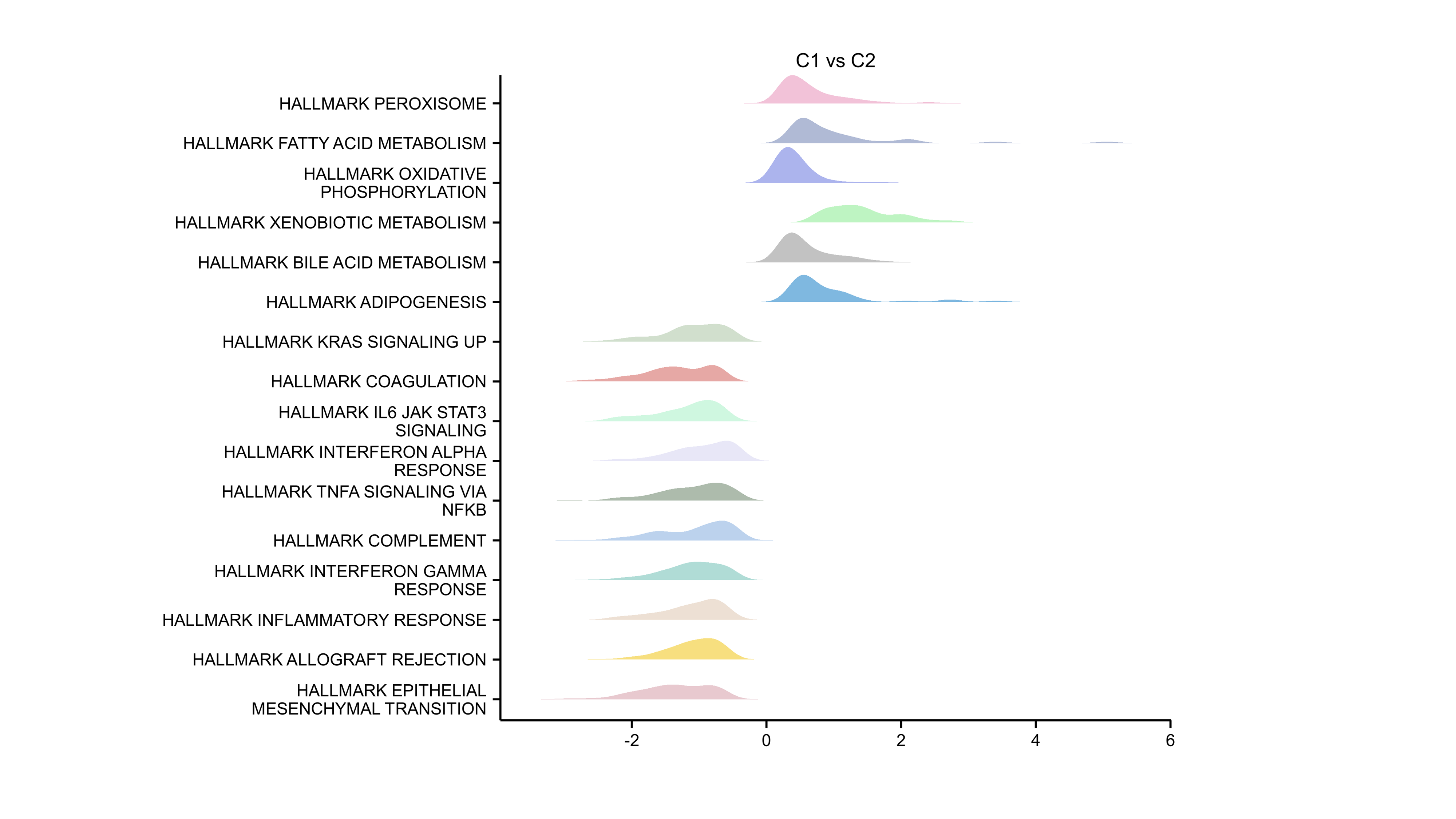

Supplement: Supplementary file 4 [file Image2.TIF]

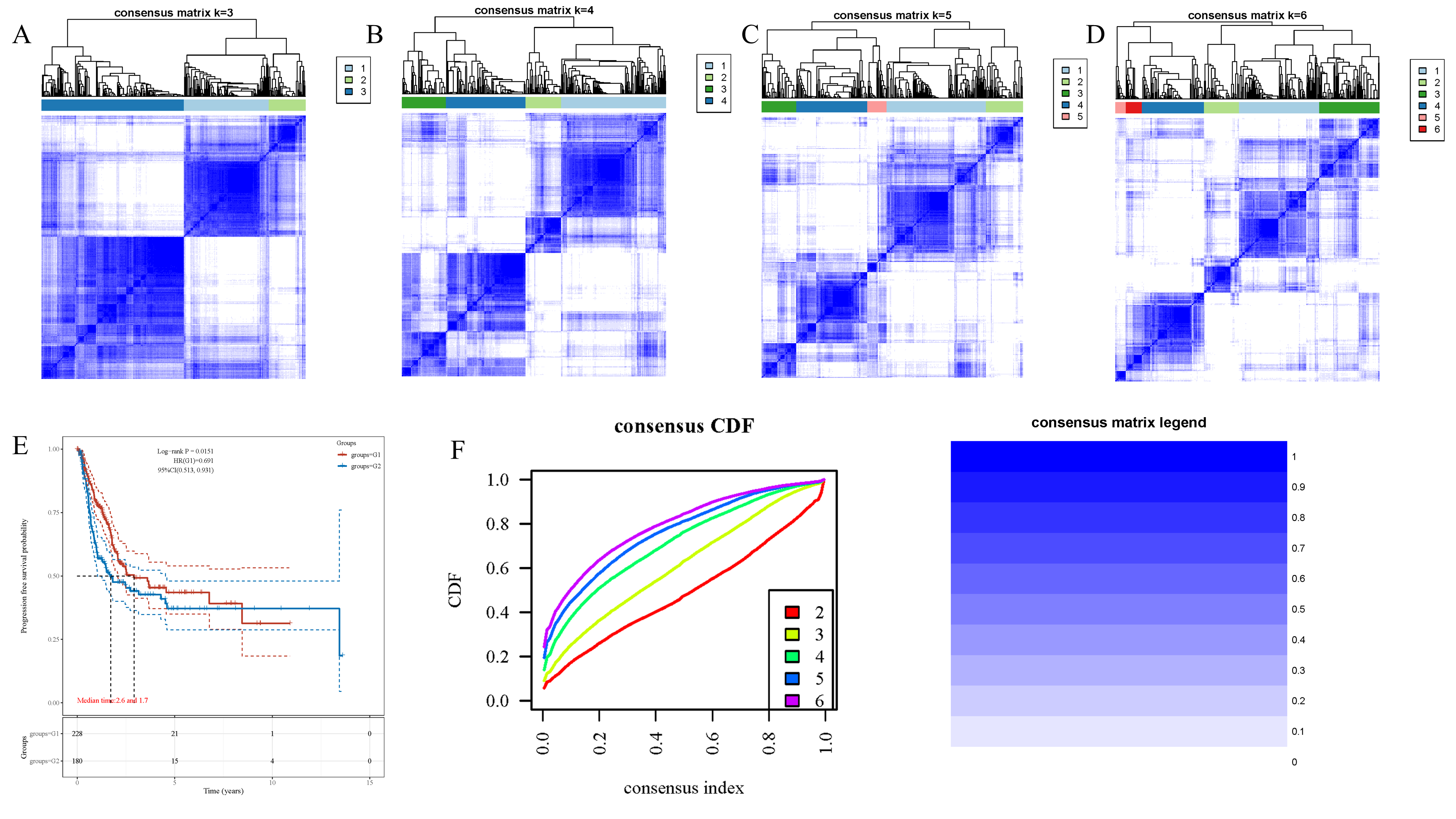

Supplement: Supplementary file 5 [file Image1.TIF]

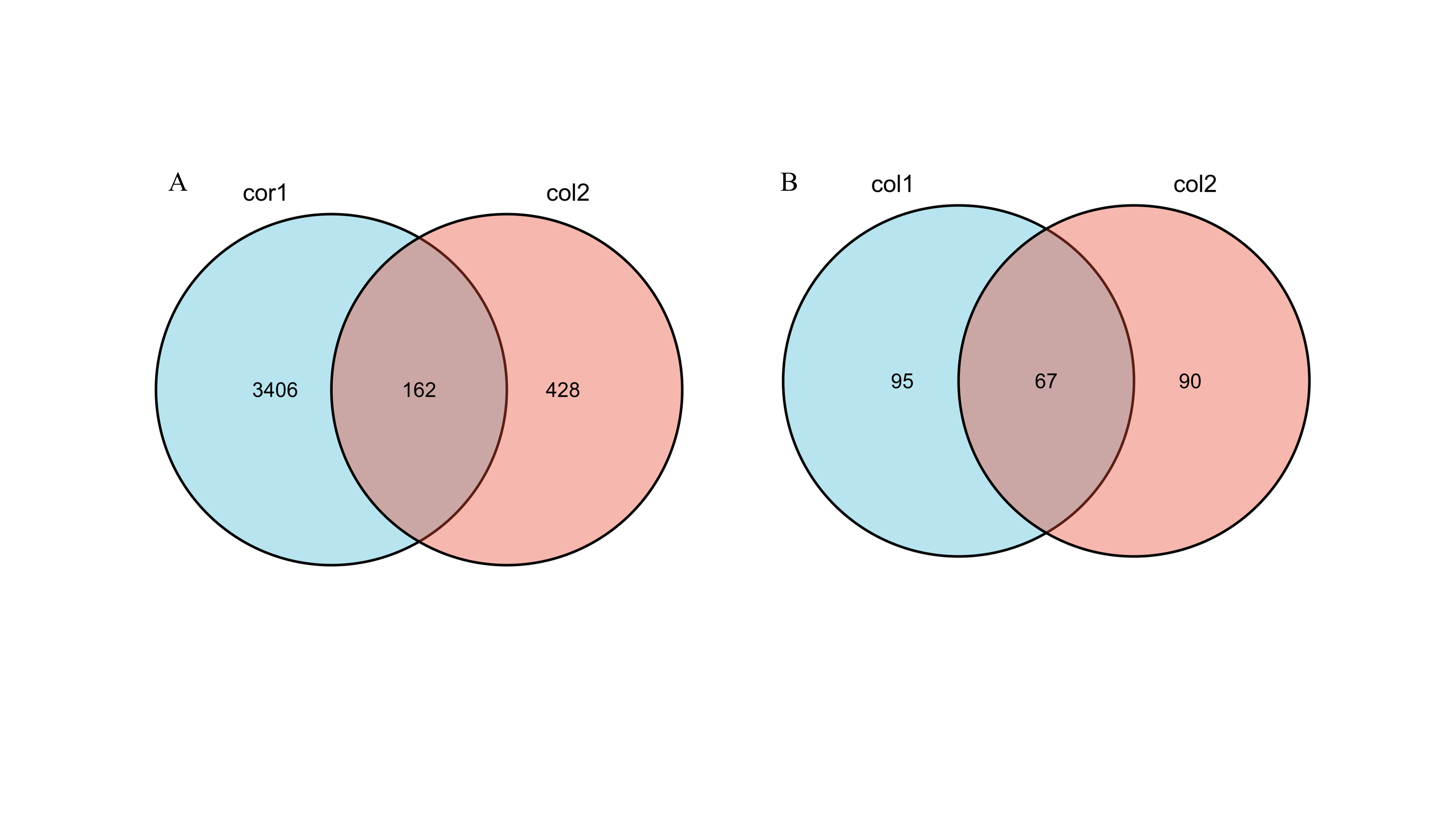

Supplement: Supplementary file 6 [file Image5.TIF]
